# Distal brachial-radial artery bypass with periarterial sympathectomy in Buerger disease: a case report

**DOI:** 10.1093/jscr/rjag361

**Published:** 2026-05-09

**Authors:** Dev Patel, Diana Mastellone, A Bobby Chhabra, Michael J Franco

**Affiliations:** Cooper Medical School of Rowan University, 401 Broadway, Camden, NJ 08103, United States; Cooper Medical School of Rowan University, 401 Broadway, Camden, NJ 08103, United States; University of Virginia School of Medicine, 1340 Jefferson Park Ave, Charlottesville, VA 22903, United States; Cooper Medical School of Rowan University, 401 Broadway, Camden, NJ 08103, United States; Department of Plastic and Reconstructive Surgery, Cooper University Hospital, 1 Cooper Plaza, Camden, NJ 08103, United States

**Keywords:** thromboangiitis obliterans, Buerger disease, upper extremity ischemia, periarterial sympathectomy

## Abstract

Thromboangiitis obliterans (TAO), or Buerger disease, is an inflammatory vasculopathy affecting small- and medium-sized vessels. Surgical options for ischemia are often limited due to the distal nature of disease and the lack of suitable bypass targets. We present a case of upper extremity ischemia secondary to TAO treated with distal brachial-to-radial artery bypass and periarterial sympathectomy. A 46-year-old woman with a history of tobacco use presented with progressive left-hand pain, numbness, and diminished distal pulses in the extremity. Angiography demonstrated occlusion of the distal brachial artery with segmental occlusions of the radial and ulnar arteries and reconstitution of the palmar arch through the interosseous artery. Revascularization and sympathectomy resulted in initial symptomatic improvement. At 3-year follow-up, the patient reported continued avoidance of tobacco and sustained improvement in symptoms. Though smoking cessation is essential, arterial reconstruction combined with periarterial sympathectomy may provide symptom relief and improved tissue perfusion in TAO.

## Introduction

Thromboangiitis obliterans (TAO), also known as Buerger disease, is a non-atherosclerotic inflammatory occlusive disease affecting small- and medium-sized arteries and veins of the extremities. The disease is strongly associated with tobacco exposure, and history or current smoking is often part of the diagnostic criteria [1]. Pathologically, TAO is characterized by inflammatory thrombus formation within the vessel lumen with relative preservation of the vessel wall architecture [1].

Patients typically present with distal extremity ischemia characterized by rest pain, digital ulceration, or gangrene [2]. Although lower extremity involvement is more common, the upper extremity may also be affected [3]. Typically the disease targets distal small arteries and moves proximally as it progresses [1]. Management is primarily centered on smoking cessation, which remains the most important intervention for halting disease progression [2]. Medical therapies including antiplatelet agents, vasodilators, and prostacyclin analogs may improve symptoms but have limited impact on long-term outcomes [4].

Surgery in TAO remains controversial, as suitable distal target vessels for revascularization are not often present [4]. Additionally, surgical revascularization in TAO is challenging due to lower patency rates, a recent study found lower patency rates in TAO patients over atherosclerosis, 77.8% versus 92.9%, respectively, and 1 year follow-up patency of 44.5% versus 85.7% [5].

The purpose of this report is to describe a case of severe upper extremity ischemia treated with distal brachial-to-radial artery bypass combined with extensive periarterial sympathectomy and to review the potential role of surgical management in select patients with TAO.

## Case report

A 46-year-old woman presented to the hand surgery clinic with progressive left hand pain and intermittent numbness. Her medical history was significant for tobacco use and a prior episode of thrombosis involving the distal brachial artery that had been treated with thrombectomy. Despite this intervention, she experienced recurrent symptoms. At the time of presentation, she reported severe rest pain involving the hand and fingers. She had recently discontinued tobacco use but had not experienced any improvement in her symptoms with cessation. Laboratory evaluation including inflammatory markers, autoimmune serologies, and hypercoagulability testing was negative. Physical examination demonstrated diminished pulses in the affected extremity and an abnormal Allen test. Despite the severity of her symptoms, there was no evidence of ulceration or gangrene.

**Figure 1:**
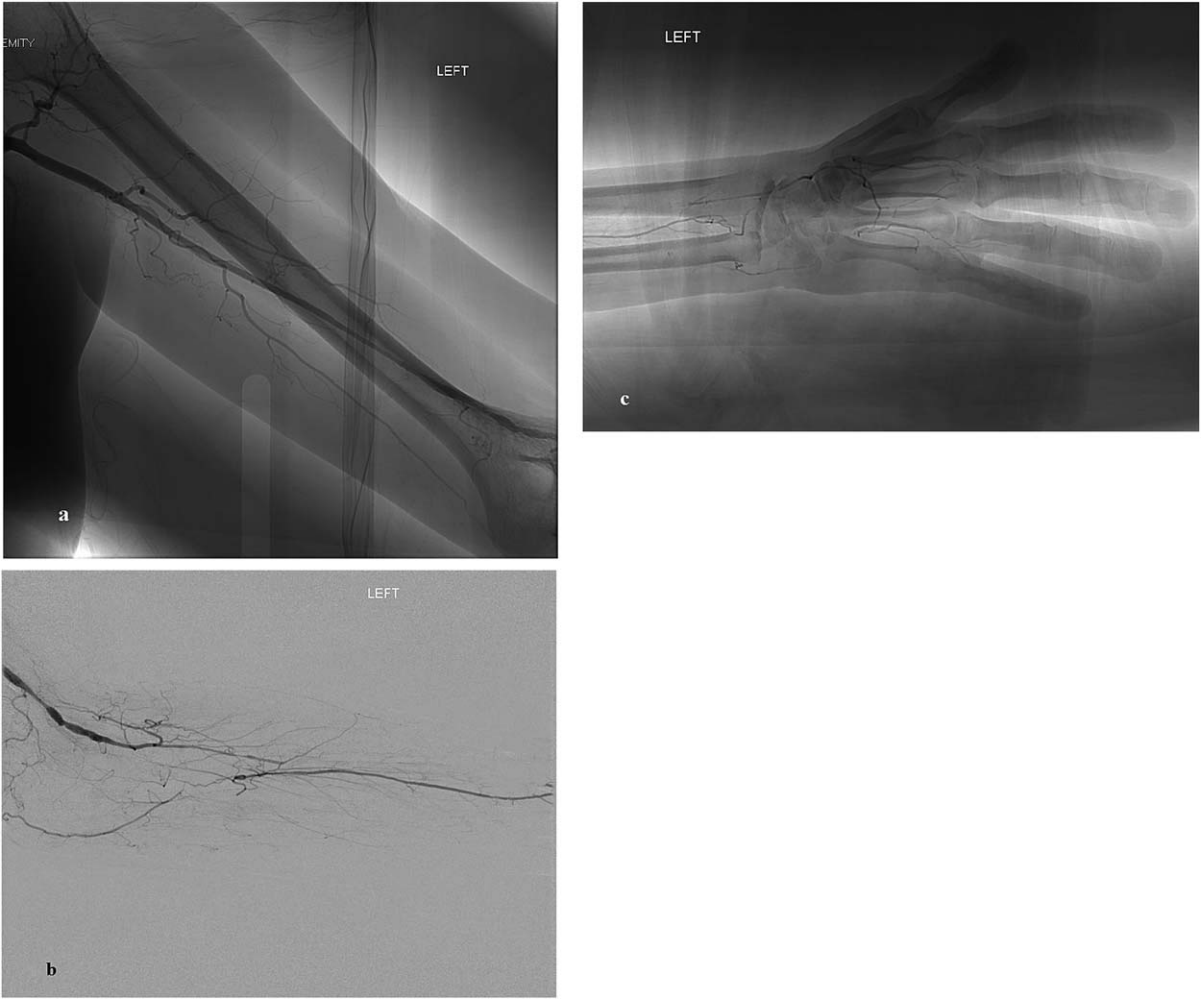
(a) Arteriogram of the left upper extremity demonstrating segmental occlusion of the distal brachial artery. (b) Arteriogram of the left upper extremity demonstrating occluded proximal radial and ulnar arteries with flow through a patent interosseous artery. (c) Arteriogram of the left upper extremity demonstrating occluded vessels in the hand with communication between the distal radial artery and interosseous artery.

Angiography demonstrated occlusion of the distal brachial artery with segmental occlusion of both the radial and ulnar arteries (Fig. 1a). Perfusion of the hand occurred through the interosseous artery with reconstitution of the palmar arch (Fig. 1b and c). Given the severity of symptoms, failure of medical management, and motivation to continue with smoking cessation, surgical intervention was planned.

A multidisciplinary approach was utilized involving both vascular and hand surgery teams. The vascular surgery team harvested a reversed saphenous vein graft using an endoscopic technique and provided proximal exposure of the brachial artery. The hand surgery team performed extensive periarterial sympathectomy involving the radial and ulnar arteries at the wrist, the radial artery in the anatomic snuffbox, the superficial palmar arch, and the common digital arteries. A distal brachial-to-distal radial artery bypass was then performed using a reversed saphenous vein graft measuring ~35 cm in length. The distal radial artery was selected as the target vessel based on angiographic findings and intraoperative Doppler signals demonstrating distal flow into the palmar arch. At the conclusion of the procedure, Doppler examination demonstrated flow through the graft and into the hand. The patient was maintained on aspirin and anticoagulation therapy postoperatively.

**Figure 2:**
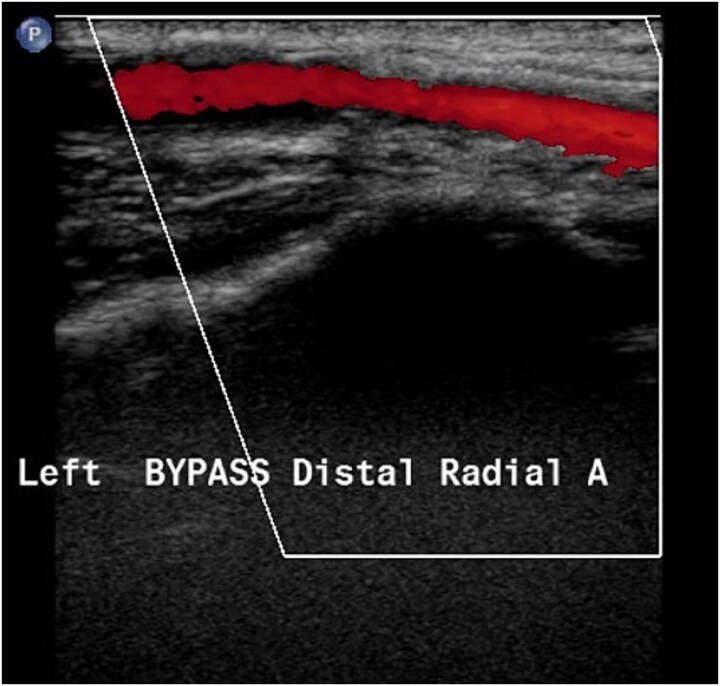
Image from duplex ultrasonography demonstrating flow through the saphenous vein bypass graft at 14 months after the second operation.

At 2-month follow-up, the patient reported significant improvement in ischemic pain and no longer required analgesics. She noted near resolution of her symptoms. Approximately 4 months following surgery, the patient returned with recurrence of hand pain. During this period, she had resumed tobacco use. Angiography revealed thrombosis of the bypass graft. After counseling and renewed efforts toward smoking cessation, the patient underwent revision bypass surgery using a long reversed saphenous vein graft harvested from the contralateral lower extremity. The distal anastomosis was performed to the radial artery distal to the previous graft site. Postoperative Doppler examination demonstrated restoration of flow through the superficial palmar arch. At 14-month follow-up, duplex ultrasonography demonstrated patency of the bypass graft (Fig. 2). At 3-year follow-up, the patient reported continued improvement in upper extremity ischemic symptoms.

## Discussion

Surgical management of TAO remains challenging due to the distal distribution and segmental nature of the disease [1]. In many cases, distal target vessels suitable for bypass are not present, limiting surgical options [4]. Nevertheless, selected patients with persistent ischemia after smoking cessation may benefit from surgical intervention. If the anatomy enables revascularization, the increased distal blood flow may provide symptomatic relief. Periarterial sympathectomy has been used as treatment for digital ischemia caused by a variety of factors, including TAO. A systematic review of studies evaluating sympathectomy for chronic digital ischemia have shown some improvement in ulcer healing and lower amputation rates; however, many studies group outcomes for TAO with atherosclerosis, making it difficult to determine the benefit of sympathectomy in TAO alone [6]. Theoretically, sympathectomy combined with bypass in TAO may have an additive effect by enhancing distal perfusion and contributing to symptomatic improvement. Although long-term patency rates for bypass grafts in TAO are variable and generally lower than those observed in other types of grafts [5], short-term increases in blood flow may be sufficient to relieve pain and promote healing.

## Conclusion

In this case, distal brachial-to-radial artery bypass combined with extensive periarterial sympathectomy resulted in meaningful improvement in ischemic symptoms. Graft thrombosis occurred following resumption of tobacco use, highlighting the critical role of smoking cessation in disease management [2]. Further study is needed to better define the role of revascularization and sympathectomy in the management of TAO.
